# InTrack project ˗ Theoretical framework, design, and methods: A study protocol

**DOI:** 10.1371/journal.pone.0283157

**Published:** 2023-03-30

**Authors:** Mabliny Thuany, Thayse Natacha Gomes, Katja Weiss, Volker Scheer, Lee Hill, Ramiro Rolim, Beat Knechtle, Marcos André Moura dos Santos

**Affiliations:** 1 Centre of Research, Education, Innovation and Intervention in Sport (CIFI2D), Faculty of Sport, University of Porto, Porto, Portugal; 2 Department of Physical Education, Post-Graduation Program of Physical Education, Federal University of Sergipe, São Cristóvão, SE, Brazil; 3 Department of Physical Education & Sports Science, University of Limerick, Limerick, Ireland; 4 Physical Activity for Health Cluster, Health Research Institute, University of Limerick, Limerick, Ireland; 5 Medbase St. Gallen Am Vadianplatz, St. Gallen, Switzerland; 6 Institute of Primary Care, University of Zurich, Zurich, Switzerland; 7 Ultra Sports Science Foundation, Pierre-Benite, France; 8 Division of Gastroenterology & Nutrition, Department of Pediatrics, McMaster University, Hamilton, Canada; 9 Research Institute of the McGill University Health Centre, Montréal, Québec, Canada; 10 Associated Postgraduate Program in Physical Education, University of Pernambuco and Federal University of Paraiba, Recife, Brazil; University of Split, CROATIA

## Abstract

Sports performance is the result of a complex interaction between individual and environmental factors. The purpose of this paper is to explain the methods used in the InTrack Project, a cross-sectional and cross-cultural project developed to investigate the variance in the performance of runners from different countries and to understand whether the differences in the performance can be explained by micro-level (athletes characteristics and proximal environment), meso-level (the distal environment that plays a relevant role on the relationships established at micro-level), and the macro-level (environmental features that shape countries characteristics). The sample will be comprised of runners, of both sexes, from four countries. Data collection will be performed in two steps: i) Individual information and ii) Country-level information. At the individual level, data will be obtained from an online survey. At the country level, characteristics data will be obtained from the secondary data available (demographic, social, and economic variables). Statistical procedures expected to be used include multilevel analysis, latent class analysis, addictive and multiplicative interaction in regression models. This wealth of information is of relevance to fill gaps regarding the existence of variables to connect different levels of information, and to provide scientific support about environmental characteristics important to predict runners’ performance within and between countries.

## Introduction

Athletes’ performance is a dynamic, non-linear, and multidimensional phenotype [1], characterized as being complex and multi-faceted. These characteristics reinforce the use of holistic approaches to better and deeper understand this phenomenon. From the set of available theories, Bronfenbrenner’s ecological systems theory (1977, 2011) was previously highlighted. Firstly presented in 1977 [2], the theory was developed as a critique of experimental psychology, and designed to provide a new approach to studying children’s development. As most of the studies were unidirectional, adopting the behavior as an outcome of the environment, the theory suggests that children’s development must be studied considering the interplay of subject-context [2]. Further, the theory proposes that variables responsible for the expression of a given behavior are derived from different levels/contexts, which are positioned in a hierarchical structure (from the closest to the furthest from the subject), interacting within and between levels.

Moving forward the borders of psychology, the ecological systems theory has been applied in sports science, supporting studies from auxology to sports performance [3, 4]. Since athlete’s performance is the result of the interaction between variables that come from different levels/environments, which can be located both close or distant from athletes, the use of ecological system theory to understand the expression of this outcome has expanded [5]. In summary, the different levels include the micro-level–intrapersonal and training characteristics, adding the proximal environment, such as coach-athlete dyad, family and friends support; the meso-level–where athletes’ direct relationships are not observed, but whose environment plays a relevant role on the relationships established at micro-level, such as club sports, sports federations; and the macro-level–environmental features that shape sports systems, including culture, economic, and demographic indicators [6–9].

The interplay of these different levels accounts for the differences in the expression of athlete’s performance intra and inter countries since athletes live under different natural, social, and cultural environments [10–12]. In other words, between-countries differences result from the interaction of different domains, including (but not limited to) the political system, economy, education, cultural factors, military systems, and living conditions (e.g., poverty levels, human development indicators) [13, 14]. As a result of these differences, it is expected to observe differences in sports performance at an international level, given that inputs (e.g., economic support, sports culture, dissemination, and access) differ between countries. For example, in some African countries, most budgets are perpetually stretched to urges, such as conflict resolution, hunger, and poverty eradication [14], meaning that notwithstanding the role of sports can play in these societies, other needs require more investments. In addition, hidden features, such as the shared attitudes and cultural values within groups can be related to sports representativeness or its absence.

Studies aiming to explain international sports success through countries’ characteristics have shown that population size, human development index (HDI), and political systems can explain half of the performance between countries [15]. The HDI–an index that comprises health, income, and formal education access–is considered one of the most important variables that, in association with cultural boundaries, foster an atmosphere of sports development [16]. A positive association was found between states HDI and the likelihood to be a soccer player in a first-division club in the Brazilian context [16, 17]. Similarly, athletes from cities with high HDI were six times more likely to become a swimmer compared to those born in cities with lower HDI [18]. Specifically, in running context, the 20 best sprinters ranked worldwide (2006 to 2016) were from countries with high HDI, while those competing in endurance events (10,000 meters or above) came from low/middle HDI countries [6]. These results were related to the training specificities and the African phenomenon, which biased the results of endurance events.

Despite the interest in understanding the complex features of athlete’s environment, research focusing on the role of country-related variables are still reduced. Due that sports performance is a “global race” [12, 19], and that athletes’ success is likely to be developed, studies investigating similarities/differences between countries are of relevance [19]. The Sports Policy factors Leading to International Sporting Success project, developed in 15 countries participants in the summer Olympic Games, showed that financial support is an input to countries’ performance (i.e., number of medals), as well as that each country operates in a unique system, considering specific environment features [20]. In addition, the authors highlighted the importance of considering cultural aspects, as well as the study of specific sports disciplines [21].

Running is considered a low-cost practice with easy access that can be performed under a diversity of geographical conditions [22]. The increment in the number of participants in running events [23, 24], and the barriers broken over the last years [25] increased the interest in understanding factors related to runners’ performance, including, but not limited to genetic parameters [26, 27], physiological [28], anthropometric [29], physical fitness [30, 31], and training characteristics [32, 33]. However, besides this athletes-centered approach, the context to which athletes belong must be considered an important factor related to their performance. For example, the dominance of Kenyan and Ethiopian athletes in long distances disciplines has been debated, which not only considers the exceptional characteristics of the athletes, but also the historical background, possibilities for economic progress, and potential for social rise and better living conditions through the sport [34]. Although these factors work as an important input for running, there is no information about the mechanisms by which macro-level characteristics affect the outputs (athletes’ performance). In this sense, cross-level interactions need to be investigated, to deeply understand what are the variables capable to connect different levels.

Taking into account within- and between- countries differences, understanding specific environments which act differently on the development of runners’ performance is helpful. This wealth of information is of relevance to fill some gaps regarding the existence of variables to connect different levels of information, that is, characteristics at a superior level that can be linked to environmental features and training opportunities at lower levels; and also to provide scientific support for public policies programs with benefits for both, individual and societal levels. In this way, the main question that guided this project is: Is there performance variance between runners from different countries? If there is, can these differences be explained by micro-, meso- and macro-level variables?

### Specific purposes

○ To verify runner’s profile differences within- and between- countries;
○ To identify if different clusters derived from economic, and social support, predict within and between countries differences in running performance;
○ To test cross-level interactions, considering additive and multiplicative effects to predict runners’ performance;
○ To verify the non-linear relationship between country-level variables and runners’ performance through the Network models.

### Conceptual model

**Fig 1:**
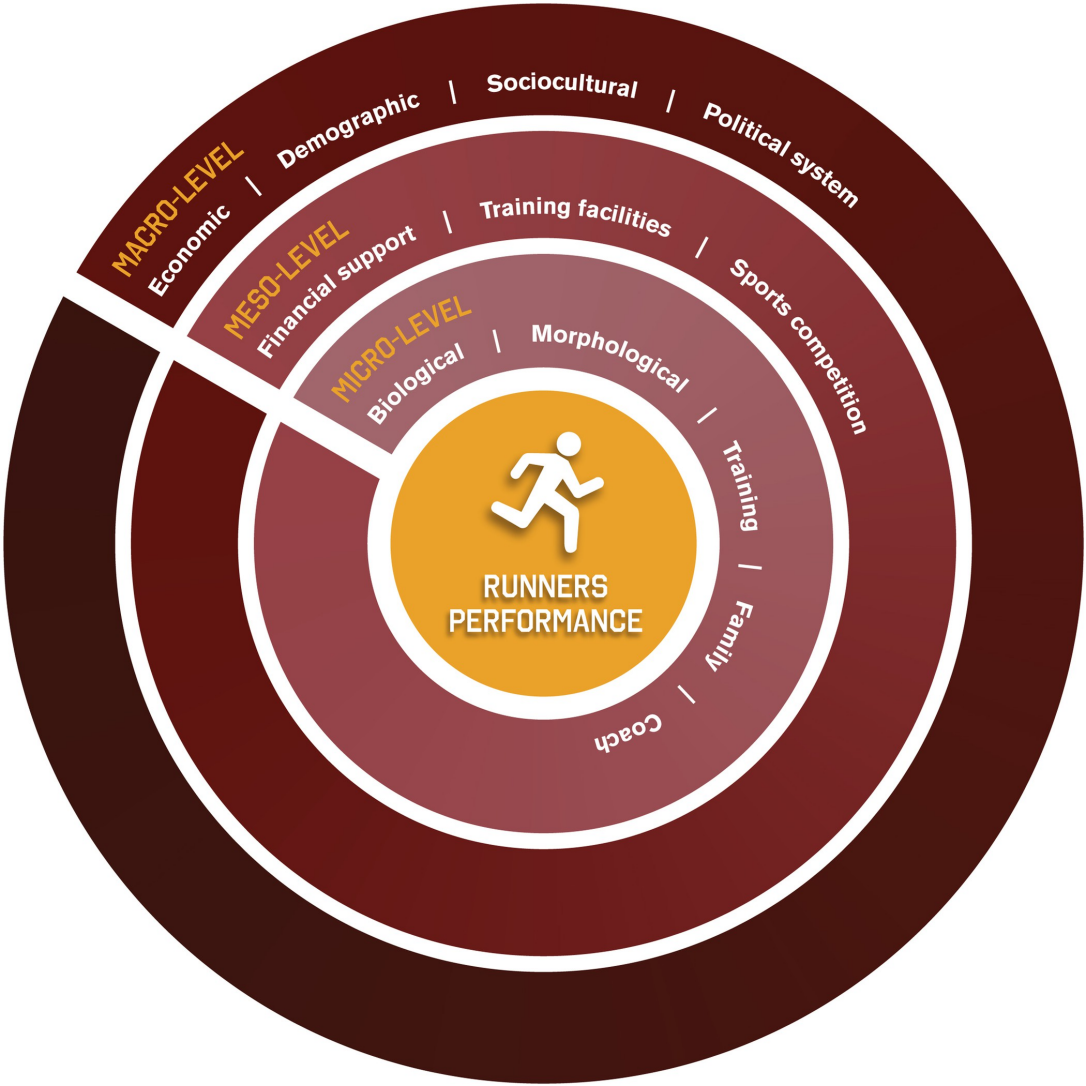
The theoretical approach of the InTrack project.

Fig 1 summarizes the conceptual approach of the project. This model was designed to indicate: *1)* the hierarchical structure between micro-, meso-, and macro-levels; *2)* the integration between different levels; and *3)* the relative importance of the variables within and between levels. The core of the figure highlights the main phenotype in which we are interested–running performance. Performance can raise some debates in the scientific context [35]. In the present project, considering the design and logistical boundaries, performance will be understood as a product (output), because as such running performance is developed based on the inputs. The nested structures consider the information/variables from different levels. At the same time, the gradient colors and the pattern model were designed based on an athletics track, which means that the further away from the core (center), the smaller the direct influence of the variables in the explanation of runners’ performance variance. In summary, these ideas highlight that different variable, at different levels, are more or less connected, and running performance behavior emerges from this interplay. The cross-sectional line highlights the interplay between levels and can be associated with a “start line” in the athletics track. Therefore, it is an unfinished model, which was not designed to be a theoretical model.

## Methods

### Design and sample

This is a cross-sectional and cross-cultural study, named InTrack project (https://www.intrackproject.com/). The sample will comprise runners, of both sexes, from different countries. The expected sample size is estimated to be at least 80 participants, in each country (sample size estimated based on the Gpower 3.1 software, considering: effect size: 0.35; α err prob: 0.05; Power: 0.95; number of predictors: 10). The study was approved by the ethics committee in the in Brazil (Federal University of Sergipe, protocol n° 5.286.914), Kenya (National Commission for Science, Technology and Innovation, NACOSTI/P/22/18904), and Portugal (Faculty of Sports, University of Porto—CEFADE 12/2022), and complies with the recommendations for the 7^th^ Revision of the Declaration of Helsinki. Participants will be informed about the risks and benefits of voluntary participation in the project. Considering that data will be collected using a web survey, for informed consent, all participants must click on the option that indicates "I have read and agree to participate in the research". Participants will receive a copy of the concordance term in the email. This procedure was approved by the ethical committee.

### Eligibility criteria

*Athletes’ eligibility criteria* ˗ runners must self-classify as a runner, age ≥18 years; have taken part in at least one official competition in the last 12 months previous to data collection; and answer the online questionnaire. Athletes that do not answer all the mandatory questions of the questionnaires (i.e., country of residence and running pace) will be excluded during the data analysis process.

*Countries’ eligibility criteria* ˗ The countries’ inclusion in the project is conditioned by the approval of the local ethics committee

### Data collection procedures

The research will occur in a virtual environment, from the participant recruitment to the data collection phases. The dissemination of the research will be carried out through contact with athletics Federations of the countries, higher education institutions, social media, personal social networks of the research team, and through prior contact with sports clubs during the years 2022/2023. After showing interest in participating in the research, a link with the questionnaire will be sent and the consent form. Only after agreeing to participate in the research, by signing the consent form, the participant will have access to the questionnaire to answer. The participant is allowed to abort the process of answering the questionnaire or abstain from answering the questions. When completing the questionnaire, the participant will receive a message indicating that the questionnaire has been completed. Within 12 months of the data collection, the personal information gathered for this study will be pseudonymized, and within/after 18 months, it will be completely anonymized. True anonymization renders information non-personal.

## Individual-level information

### Microsystem-level

Athletes’ information will be obtained through a web survey, shared through the Google Forms platform. This strategy has been largely used in different research fields [36, 37], and cross-cultural research showed equivalence between paper and web-survey modes of administration [38]. The questionnaire was developed by the authors based on the theoretical framework of the project [2, 39] and previous instruments [40, 41].

The questionnaire comprises five domains and 37 items, and participants will spend approximately 15 minutes completing it. The questionnaire provides information about runner identification (age; sex); anthropometric variables (body height; body mass); sociodemographic profile (country of current living; monthly income; educational level; marital status); training characteristics (volume, duration, and frequency/week; sessions/day; practice time; running pace); involvement in an official running event; motivation for the practice; relationship with coaches; and perception about contextual support (family, friends, coach, training facilities). Mandatory questions include information about the country of residence and running pace (outcome variable).

The questionnaire’s psychometric quality will be tested through evaluation by experts and also by a pilot test among Brazilian runners. Items and domains will be assessed considering “content”, “objectivity”, “clarity”, “readability”, and “understanding of the content” [42]. Following, for its use in non-Portuguese speakers’ countries, a translation, followed by a back translation, will be performed. A cultural equivalence is possible to be performed.

## Country-level information

### Mesosystem information

Mesosystem information will be obtained from free access web pages and documents for each country. The information about the existence of programs for the selection and development of sports talent, the number of high-performance athletes in the ranking of the sports disciplines at national and international (world) levels, the number of sports clubs, the number of high-performance competitions and sports investment in the modality will be gathered. Additional information can be obtained.

### Macro-level information

Demographic, economic, and sports financial support data will be obtained from free access web pages and official documents with open and unrestricted access from each country (National Institute of Statistics of the countries; National datasets). The following information will be gathered: a) population size and density; b) HDI; c) gross domestic product; d) per capita income; e) annual sports investment; f) the number of high-performance athletes in the sport’s ranking at international (world) level; g) cultural aspects. Cultural information refers to the six dimensions for Hofstede [43]–power distance, individualism, masculinity, uncertainty avoidance, long-term orientation, and indulgence.

### Expected statistical procedures

**Fig 2:**
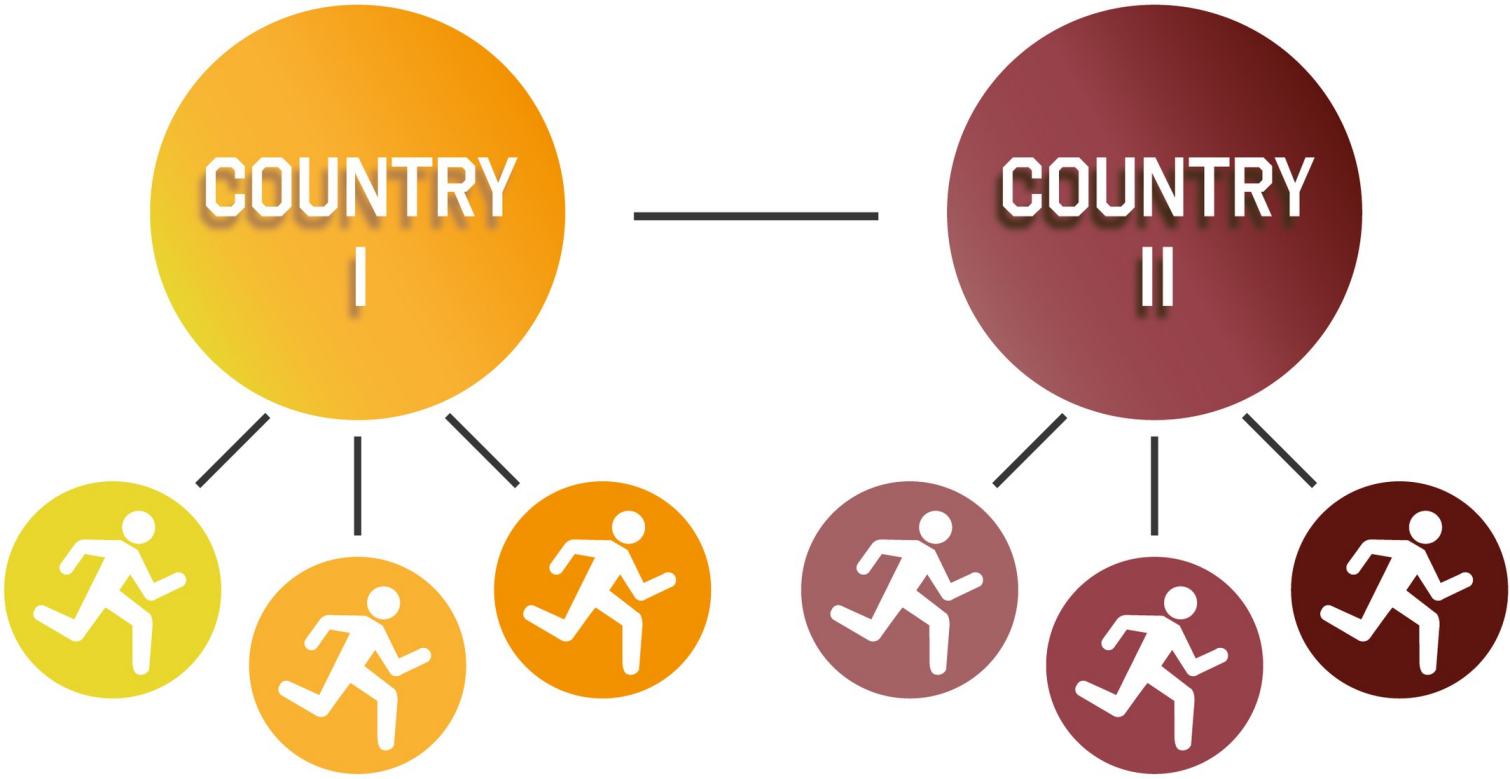
InTrack project data structure.

Descriptive information will be presented in the five-number summary in statistics (minimum value, first and third quartile, the median, and maximum values), mean (standard deviation), and frequencies (%). Given the nested structure of the data, for the main question of the InTrack project, the multilevel analysis will be computed. Multilevel analysis, also named hierarchical linear models, linear mixed-effect models, mixed models, or nested data models [44], considers the data organization at different levels. The first important information includes the intraclass correlation coefficient, which measures the total variance that can be attributed to group differences [45]. If we confirm this assumption, two levels will be estimated–level one (runners) and level two (countries) (Fig 2). The main idea is to estimate the statistically significant differences between runners from different countries; and to determine the performance variance within- and between- countries, as well as individual and environmental factors associated with running performance.

Latent Class Analysis (LCA) will be used for the secondary aims. LCA is a statistical person-centered procedure, used to cluster subgroups that share specific characteristics [46]. LCA uses categorical variables, and in the present project, we intend to use this procedure to verify how athletes are nested within- and between- countries based on different variables, such as economic aspects, training characteristics, and environmental perception, as well as to find if there is a class with the highest probability to present better performance. The confidence interval will be fixed at 95%.

In addition, a Network analysis will be used to identify the relationship between country-level variables and runners’ performance. The Network analysis is a set of integrated techniques to detail relations between variables/subjects and to analyse the structures that emerge from these relations [47]. For the present project, this analysis will allow the understanding of the non-linear interactions between variables presented in our conceptual model and to identify variables that are hubs (i.e., “bridges”) between micro and macro-levels. In addition, multiple regression models will be performed, considering both addictive and multiplicative effects to predict runners’ performance within and between countries.

### Broadcasting findings

We will disseminate trial results to all interested (e.g., participants, stakeholders, partners). Results will be submitted for publication in peer-reviewed journals and presented at scientific events (e.g., congress, meetings, and symposiums). Authorship of publications will be based on contributions toward design, data collection, analysis, and manuscript writing. All participants’ data will be anonymized (i.e., encrypted) to protect confidentiality. The project and the scientific articles will be developed following the guidelines of Improving the quality of Web surveys: the Checklist for Reporting Results of Internet E-Surveys [48].

## Discussion

Moving beyond the idea that athletes are not randomly distributed between different places, the shared values within a population, the national pride, and sports cultural values present important significance for athletes’ commitment to training. For the present project, we do not neglect the importance of genetic, physiological, psychological, and dietetic variables that influence runners’ performance [49, 50]; instead, we are using different lenses to understand this complex phenomenon. In this sense, the project presents weaknesses, threats, strengths, and opportunities that must be highlighted.

### Weakness, threats, strengths, and opportunities

The most important weakness is related to the research design. In cross-cultural studies, the risk of noise needs to be considered in different steps of the project, starting with the unit of analysis. The unit of analysis defines the scope of the research design. For the present study, the unit of analysis is the countries. Although we assume that countries are different, differences in cultural, economic, and social aspects also are verified within countries, especially in countries with higher populations and territories. In this sense, future findings generalization needs to be considered carefully.

Methodological issues include data collection (i.e., procedures and instruments), which is expectable to be standardized between countries. For the present study, dissemination of the research will be carried out using the same procedure in all countries. However, the use of a questionnaire for data collection is prone to bias since some inequivalence can be seen between countries. This means that a given question can be understood differently by participants from different countries (and even from the same country) due to different backgrounds and perspectives. To reduce this bias, the development of the questionnaire was based on the theoretical framework, considering the opinions of different researchers, from the different countries that are part of the project. In addition, statistical procedures to test the internal consistency of the instrument will be performed. Is important to refer that the project was not designed to provide representative data from each participating country. Instead, the methodological approach was designed to allow data collection in different countries, using a low-cost strategy. However, we are conscious of the limitations of using this method.

Another important limitation is the “black box problem” since we are unable to understand the mechanism between inputs and outputs. Therefore, we intend to explore the moderator role of variables situated at the meso and micro levels that can be related to training commitment and runners’ performance. A further problem is an ecological fallacy, defined as a problem of confounding [51]. This means that individual estimates are based on aggregated data for a group, which implies the consequence of the inferences—for example, cultural values obtained at the country level do not mean that all runners share the same trait/behavior. Last, but not least, we must be careful in investigating different countries. Culture, values, beliefs, and the countries’ pro-community atmosphere are difficult to measure. However, when comparing countries we are also adopting “hidden characteristics” as control variables. In addition, as we are using secondary data to refer meso and macro-level, the potential to explore variables is associated with the availability and updated information.

Threats of the project include the use of an online questionnaire for data collection. Although widely used, this strategy can present some barriers to access, and reduce the return rate, limiting the sample size and generalization of the findings. In addition, a body of evidence is available about the bias associated with self-reported information. These biases include over- and under-reporting information, social expectations, recall, and confirmation bias [52]. However, the bias can be lower for training characteristics, since about eight out of 10 runners [53] use devices to record and monitor training. Even though using a cross-sectional approach, we are not considering the dynamic characteristics of the performance.

Besides the weaknesses and threats, our strengths include the use of information on a hierarchical level and the exploration of possible relationships between these different levels with athletes’ performance. Results obtained from the project can advance the understanding of environmental factors associated with performance. Comparing countries may provide insights into understanding the differences in international sports success and engagement. The last important aspect may border on hyperbole, but the positive impact of sports on human development must not be underestimated.

## References

[1] Balague N, Torrents C, Hristovski R, Davids K, Araújo D. Overview of complex systems in sport. Journal of Systems Science and Complexity. 2013;26(1):4–13. doi: 10.1007/s11424-013-2285-0

[2] Bronfenbrenner U. Toward an experimental ecology of human development. American Psychologist. 1977;32(7). doi: 10.1037/0003-066X.32.7.513.

[3] Fathirezaie Z, Abbaspour K, Badicu G, Zamani Sani SH, Nobari H. The Effect of Environmental Contexts on Motor Proficiency and Social Maturity of Children: An Ecological Perspective. Children (Basel, Switzerland). 2021;8(2):157. doi: 10.3390/children8020157 33669542 PMC7923079

[4] Poulus DR, Coulter TJ, Trotter MG, Polman R. A qualitative analysis of the perceived determinants of success in elite esports athletes. J Sports Sci. 2022;40(7):742–53. doi: 10.1080/02640414.2021.2015916 34930102

[5] Vella SA, Cliff DP, Okely AD. Socio-ecological predictors of participation and dropout in organised sports during childhood. Int J Behav Nutr Phys Act. 2014;11(62). doi: 10.1186/1479-5868-11-62 24885978 PMC4041056

[6] Santos P, Sousa C, da Silva Aguiar S, Knechtle B, Nikolaidis P, Sales M, et al. Human Development Index and the frequency of nations in Athletics World Rankings. Sport Sci Health. 2019;15(2):393–8. doi: 10.1007/s11332-019-00529-1.

[7] Thuany M, Gomes TN, Hill L, Rosemann T, Knechtle B, Almeida MB. Running Performance Variability among Runners from Different Brazilian States: A Multilevel Approach. Int J Environ Res Public Health. 2021;18(7). Epub doi: 10.3390/ijerph18073781 . 33916357 PMC8038602

[8] Campbell E, Irving R, Poudevigne M, Dilworth L, McFarlane S, Ismail O, et al. Contextual factors and sporting success: The relationship between birth date and place of early development on the progression of Jamaican track and field athletes from junior to senior level. PLoS One. 2019;14(12):e0227144. Epub doi: 10.1371/journal.pone.0227144 . 31881050 PMC6934301

[9] Wicker P, Hallmann K, Breuer C. Micro and macro level determinants of sport participation. Sport, Business and Management: An International Journal of Behavioral Nutrition and Physical Activity. 2012;2(1):51–68. doi: 10.1108/20426781211207665

[10] De Bosscher V, De Rycke J. Talent development programmes: a retrospective analysis of the age and support services for talented athletes in 15 nations. European Sport Management Quarterly. 2017;17(5):590–609. PubMed PMID: 125601755.

[11] Bosscher VD, Knop PD, Bottenburg MV, Shibli S, Bingham J. Explaining international sporting success: An international comparison of elite sport systems and policies in six countries. Sport Management Review. 2009;12(3):113–36. doi: 10.1016/j.smr.2009.01.001

[12] Bosscher V, Bingham J, Shibli S, Bottenburg Mv, Knop P. The global sporting arms race—an international comparative study on sports policy factors leading to international sporting success. Oxford: Meyer & Meyer Sport; 2008.

[13] De Bosscher V, Shibli S, Westerbeek H, Bottenburg Mv. Successful elite sport policies: An international comparison of the Sports Policy factors Leading to International Sporting Success (SPLISS 2.0) in 15 nations. United Kingdom: Meyer & Meyer Sports (UK) Ltd.; 2015. 402 p.

[14] Mkumbuzi NS, Chibhabha F, Zondi PC. Out of sight, out of mind: the invisibility of female African athletes in sports and exercise medicine research. Br J Sports Med. 2021;55(21):1183–4. doi: 10.1136/bjsports-2021-104202 33975826

[15] Bohme MTS, Bastos FC. Esporte de alto rendimento: fatores críticos de sucesso—gestão—identificação de talentos. 1 ed. São Paulo: Phorte; 2016. 360 p.

[16] Costa IT, Cardoso FSL, Garganta J. O Índice de Desenvolvimento Humano e a data de nascimento podem condicionar a ascensão de jogadores de Futebol ao alto nível de rendimento? Motriz. 2013;19(1):34–45.

[17] Teoldo I, Cardoso F. Talent map: how demographic rate, human development index and birthdate can be decisive for the identification and development of soccer players in Brazil. Science and Medicine in Football. 2021;5(4):293–300. doi: 10.1080/24733938.2020.1868559 35077299

[18] Gomes-Sentone R, Lopez-Gi JF, Caetano CI, Cavichioll FR. Relationship between human development index and the sport results of Brazilian swimming athletes. JHSE. 2019;14(5):S2009–S18. doi: 10.14198/jhse.2019.14.Proc5.22

[19] Winand M. The Global Sporting Arms Race. An International Comparative Study on Sports Policy Factors Leading to International Sporting Success (SPLISS). European Sport Management Quarterly. 2010;10(5):613–5. doi: 10.1080/16184742.2010.524242

[20] Bosscher VD, Shibli S, Westerbeek H, Bottenburg Mv. Successful elite sport policies: An international comparison of the Sports Policy factors Leading to International Sporting Success (SPLISS 2.0) in 15 nations. Verlag MM, editor. United Kingdom: Meyer & Meyer Sports (UK) Ltd.; 2015. 402 p.

[21] De Bosscher V, Shibli S, Westerbeek H, van Bottenburg M. Convergence and Divergence of Elite Sport Policies: Is There a One-Size-Fits-All Model to Develop International Sporting Success? Journal of Global Sport Management. 2016;1(3–4):70–89. doi: 10.1080/24704067.2016.1237203

[22] Hulteen RM, Smith JJ, Morgan PJ, Barnett LM, Hallal PC, Colyvas K, et al. Global participation in sport and leisure-time physical activities: A systematic review and meta-analysis. Preventive Medicine. 2017;95:14–25. doi: 10.1016/j.ypmed.2016.11.027. 27939265

[23] Rizzo N. Running Boom: 28.76% of runners started during the pandemic 2021 [cited 2021 01st Jun]. Available from: https://runrepeat.com/new-pandemic-runners.

[24] RunRepeat. Marathon Statistics 2019 Worldwide Online2020 [cited 2020 07 March]. Available from: https://runrepeat.com/research-marathon-performance-across-nations.

[25] Snyder KL, Hoogkamer W, Triska C, Taboga P, Arellano CJ, Kram R. Effects of course design (curves and elevation undulations) on marathon running performance: a comparison of Breaking 2 in Monza and the INEOS 1:59 Challenge in Vienna. J Sports Sci. 2021;39(7):754–9. Epub doi: 10.1080/02640414.2020.1843820 . 33176588

[26] Puthucheary Z, Skipworth JR, Rawal J, Loosemore M, Someren KV, Montgomery HE. Genetic influences in sport and physical performance. Sports Med. 2011;41:845–59. doi: 10.2165/11593200-000000000-00000 21923202

[27] Joyner MJ. Genetic Approaches for Sports Performance: How Far Away Are We? Sports Med. 2019;49(Suppl 2):199–204. doi: 10.1007/s40279-019-01164-z 31691930 PMC6901428

[28] Degens H, Stasiulis A, Skurvydas A, Statkeviciene B, Venckunas T. Physiological comparison between non-athletes, endurance, power and team athletes. Eur Jour of App Phys. 2019;119(6). doi: 10.1007/s00421-019-04128-3 30919126

[29] Mooses M, Hackney AC. Anthropometrics and body composition in east African runners: potential impact on performance. International Journal of Sports Physiology and Performance. 2017;12(4):422–30. doi: 10.1123/ijspp.2016-0408 27631418

[30] Nikolaidis PT, Coso JD, Knechtle B. Muscle strength and flexibility in male marathon runners: The role of age, running speed and anthropometry. Front Physiol. 2019;10:1301. doi: 10.3389/fphys.2019.01301 31681011 PMC6805725

[31] Nikolaidis PT, Rosemann T, Knechtle B. Force-velocity characteristics, muscle strength, and flexibility in female recreational marathon runners. Front Physiol. 2018;9. doi: 10.3389/fphys.2018.01563 30450057 PMC6224357

[32] Casado A, González-Mohíno F, González-Ravé JM, Foster C. Training Periodization, Methods, Intensity Distribution, and Volume in Highly Trained and Elite Distance Runners: A Systematic Review. Int J Sports Physiol Perform. 2022;17(6):820–33. doi: 10.1123/ijspp.2021-0435 35418513

[33] Fokkema T, Damme AAv, Fornerod MWJ, Vos R, Bierma-Zeinstra SMA, Middelkoop Mv. Training for a (half-)marathon: Training volume and longest endurance run related to performance and running injuries. Scandinavian Journal of Medicine & Science in Sports. 2020;30:1692–704. doi: 10.1111/sms.13725 32421886 PMC7496388

[34] Bale J, Sang J. Kenyan Running: Movement Culture, Geography and Global Change. 1st ed: Routledge; 1996.

[35] Raysmith BP, Jacobsson J, Drew MK, Timpka T. What Is Performance? A Scoping Review of Performance Outcomes as Study Endpoints in Athletics. Sports. 2019;7(3):66. doi: 10.3390/sports7030066 30884863 PMC6473619

[36] Wicker P, Dallmeyer S, Breuer C. Elite Athlete Well-Being: The Role of Socioeconomic Factors and Comparisons With the Resident Population. Journal of Sport Management. 2020;34(4):341–53. doi: 10.1123/jsm.2019-0365

[37] Wirnitzer K, Boldt P, Wirnitzer G, Leitzmann C, Tanous D, Motevalli M, et al. Health status of recreational runners over 10-km up to ultra-marathon distance based on data of the NURMI Study Step 2. Sci Rep. 2022;12(1):10295. Epub doi: 10.1038/s41598-022-13844-4 . 35717392 PMC9206639

[38] De Beuckelaer A, Lievens F. Measurement Equivalence of Paper-and-Pencil and Internet Organisational Surveys: A Large Scale Examination in 16 Countries. Applied Psychology. 2009;58(2):336–61. doi: 10.1111/j.1464-0597.2008.00350.x.

[39] Bronfenbrenner U. Bioecologia do desenvolvimento humano: tornando os seres humanos mais humanos. Porto Alegre: Artmed; 2011. 310 p.

[40] Thuany M, Gomes TN, Almeida MB. Validação de um instrumento para caracterização e verificação de fatores associados ao desempenho de corredores de rua. Scientia Plena. 2020;16(3):1–7. doi: 10.14808/sci.plena.2020.032801

[41] Oliveira ETd. Características e fatores associados aos corredores de Aracaju. São Cristóvão, Sergipe: Universidade Federal de Sergipe; 2015.

[42] Varanda SS, Benites LC, Neto SdS. O processo de validação de instrumentos em uma pesquisa qualitativa em Educação Física. Motrivivência. 2019;31(57). doi: 10.5007/2175-8042.2019e53877

[43] Hofstede Insights. Country comparison 2022 [cited 2022 23 August]. Available from: https://www.hofstede-insights.com/country-comparison/the-usa/.

[44] Hox J. The basic two level regression model: introduction. In: Associates LE, editor. Multilevel analysis: tecniques and applications. Mahway, New Jersey 2002. p. 304.

[45] Maia JA, Lopes VP, Silva RG, Seabra A, Ferreira JV, Cardoso MV. Modelação hierárquica ou multinível. Uma metodologia estatística e um instrumento útil de pensamento na investigação em Ciências do Desporto. Revista Portuguesa de Ciências do Desporto. 2003;3(1):92–107.

[46] Weller BE, Bowen NK, Faubert SJ. Latent Class Analysis: A Guide to Best Practice. Journal of Black Psychology. 2020;46(4):287–311. doi: 10.1177/0095798420930932

[47] Hevey D. Network analysis: a brief overview and tutorial. Health Psychology and Behavioral Medicine. 2018;6(1):301–28. doi: 10.1080/21642850.2018.1521283 34040834 PMC8114409

[48] Eysenbach Gunther. Improving the Quality of Web Surveys: The Checklist for Reporting Results of Internet E-Surveys (CHERRIES). J Med Internet Res. 2004;6(3). doi: 10.2196/jmir.6.3.e34 15471760 PMC1550605

[49] Alvero-Cruz JR, Carnero EA, García MAG, Alacid F, Correas-Gómez L, Rosemann T, et al. Predictive performance models in long-distance runners: A narrative review. International Journal of Environmental Research and Public Health. 2020;17. 33182485 10.3390/ijerph17218289 PMC7665126

[50] Coso JD, Moreno V, Gutiérrez-Hellín J, Ruíz-Moreno C, Aguilar-Navarro M, Baltazar-Martins G, et al. ACTN3 R577X genotype and exercise phenotypes in recreational marathon runners. Genes. 2019;10(413):1–15. doi: 10.3390/genes10060413 31146466 PMC6627880

[51] Schwartz S. The fallacy of the ecological fallacy: the potential misuse of a concept and the consequences. Am J Public Health. 1994;84(5):819–24. doi: 10.2105/ajph.84.5.819 8179055 PMC1615039

[52] Althubaiti A. Information bias in health research: definition, pitfalls, and adjustment methods. J Multidiscip Healthc. 2016;9:211–7. doi: 10.2147/JMDH.S104807 27217764 PMC4862344

[53] Janssen M, Scheerder J, Thibaut E, Brombacher A, Vos S. Who uses running apps and sports watches? Determinants and consumer profiles of event runners’ usage of running-related smartphone applications and sports watches. PLoS One. 2017;12(7):e0181167. doi: 10.1371/journal.pone.0181167 28732074 PMC5521773

